# Vagotomy Reduces Insulin Clearance in Obese Mice Programmed by Low-Protein Diet in the Adolescence

**DOI:** 10.1155/2017/9652978

**Published:** 2017-08-30

**Authors:** Camila Lubaczeuski, Luciana Mateus Gonçalves, Jean Franciesco Vettorazzi, Mirian Ayumi Kurauti, Junia Carolina Santos-Silva, Maria Lúcia Bonfleur, Antonio Carlos Boschero, José Maria Costa-Júnior, Everardo Magalhães Carneiro

**Affiliations:** ^1^University of Campinas (UNICAMP), Campinas, SP, Brazil; ^2^State University of Western Paraná (UNIOESTE), Cascavel, PR, Brazil

## Abstract

The aim of this study was to investigate the effect of subdiaphragmatic vagotomy on insulin sensitivity, secretion, and degradation in metabolic programmed mice, induced by a low-protein diet early in life, followed by exposure to a high-fat diet in adulthood. Weaned 30-day-old C57Bl/6 mice were submitted to a low-protein diet (6% protein). After 4 weeks, the mice were distributed into three groups: LP group, which continued receiving a low-protein diet; LP + HF group, which started to receive a high-fat diet; and LP + HFvag group, which underwent vagotomy and also was kept at a high-fat diet. Glucose-stimulated insulin secretion (GSIS) in isolated islets, ipGTT, ipITT, in vivo insulin clearance, and liver expression of the insulin-degrading enzyme (IDE) was accessed. Vagotomy improved glucose tolerance and reduced insulin secretion but did not alter adiposity and insulin sensitivity in the LP + HFvag, compared with the LP + HF group. Improvement in glucose tolerance was accompanied by increased insulinemia, probably due to a diminished insulin clearance, as judged by the lower C-peptide : insulin ratio, during the ipGTT. Finally, vagotomy also reduced liver IDE expression in this group. In conclusion, when submitted to vagotomy, the metabolic programmed mice showed improved glucose tolerance, associated with an increase of plasma insulin concentration as a result of insulin clearance reduction, a phenomenon probably due to diminished liver IDE expression.

## 1. Introduction

It has been proposed that low or high calorie intake by mothers and fathers is associated with disruption of glucose-insulin homeostasis in their offspring [1]. The mice were kept on a low-protein diet early in life and fed on a control diet, during adulthood, and also display a catch-up growth associated with glucose intolerance [2]. Indeed, economic improvements in developing countries, during recent decades, have placed human subjects into similar conditions. In these subjects, the intake of a normal- or high-calorie diet in adulthood, after a period of calorie restriction early in life, increases the risk to develop metabolic diseases [3, 4]. These early environmental situations are known as predictive adaptive response or thrifty phenotype hypothesis, postulated by Hales and Barker at 1992 [1].

We have shown that mice fed on a low-protein diet in adolescence, followed by a high-fat diet during adulthood, develop glucose intolerance, insulin resistance, and reduced insulin secretion, compared to those fed on a high-fat diet during the whole experimental period [5]. This indicates that the metabolic programming, induced by malnutrition in early life, impairs insulin-glucose homeostasis to a greater extent than obesity per se. In addition, malnourished and obese mice may display injuries to the hypothalamic neurons, which control energy intake and expenditure [6]. Glucose-intolerant mice, exposed to a low-protein diet early in life and a control diet in adulthood, also show increased vagal activity, suggesting the participation of the parasympathetic nervous system upon glucose homeostasis [2].

Metabolic programming can be explained by the Developmental Origins of Health and Diseases (DOHaD) concept that describes through several studies how early environmental factors, such as nutrition, which can induce physiological changes in fetal, neonatal, adolescence, and adult individuals, leading to a program to long-term postnatal consequences [7–9].

Thus, we sought to explore the effect of subdiaphragmatic vagotomy on insulin sensitivity, secretion, and degradation in metabolic programmed mice, induced by a low-protein diet early in life, followed by exposure to a high-fat diet in adulthood.

## 2. Materials and Methods

### 2.1. Animals

All animal experiments were carried out in accordance with the protocols approved by the Animal Care and Use Committee of the University of Campinas (UNICAMP) (approval number: 3379-1). Male C57Bl/6 mice were obtained from the UNICAMP and maintained at 22 ± 1°C in a 12 h light-dark cycle. Thirty-day-old mice were fed on a normal protein diet (14% protein) (NP group) or a low-protein diet (6% protein) (LP group) during 4 weeks. After, LP mice were distributed into three groups: LP, which was kept with a low-protein diet; LP + HF, which started to receive a high-fat diet (35% fat) during 8 weeks; and LP + HFvag, which was submitted to vagotomy and also started to receive a high-fat diet during 8 weeks. The diet compositions were described in a previous study [10].

### 2.2. Subdiaphragmatic Vagotomy Procedure

At 4 weeks after a low-protein diet consumption, LP + HF mice were submitted to subdiaphragmatic truncal vagotomy (LP + HFvag group) or sham operation (LP + HF). For this procedure, 12 h fasted mice were anesthetized with a mixture of ketamine and xylazine (0.06 and 0.02 mg/g via i.p., resp.; Vetbrands®, Paulínia, SP, BRA). Subsequently, the stomach and esophagus were exteriorized from the peritoneal cavity, and both, dorsal and subdiafragmatic vagal trunk, were separated from the esophagus and cut off. Sham-operated mice underwent the same procedures, but the vagus nerve was kept intact. At the end of the experimental period, to confirm subdiaphragmatic vagotomy, stomach food retention from all groups of mice was evaluated by the ratio between the stomach weight per body weight (BW), according to previous study [11–13].

### 2.3. Intraperitoneal Glucose and Insulin Tolerance Test

For the intraperitoneal (ip) glucose tolerance test (ipGTT), mice were fasted overnight (12 h) and a basal blood sample was harvested from the tail tip (*t* = 0 min). Mice received an ip administration of 2 g/kg glucose (Labsynth, Sao Paulo, Brazil) dissolved in saline solution (0.9% NaCl wt/vol), and additional blood samples were recorded at 15, 30, 60, and 120 min. Glucose was recorded using a handheld glucometer (Accu-Chek Performa II, Roche Diagnostics, Switzerland). For the ip insulin tolerance test (ipITT), mice were fasted for 2 hours and an ip insulin (Humulin R, Eli Lilly, Indianapolis, USA) load (1 U/kg) was administered. Blood was taken immediately before insulin injection (*t* = 0 min) and at the times 3, 6, 9, 12, 15, 18, and 21 min via tail snip using a handheld glucometer. Glucose disappearance rate (*K*_ITT_) was calculated as previously described [14, 15].

### 2.4. Insulin Clearance

During the ipGTT, blood samples were collected from the tail tip, before glucose load (*t* = 0) and 15 and 60 min after glucose administration, and placed into microtubes containing anticoagulant heparin. The tubes were centrifuged at 1100*g*, 15 min, 4°C, and the plasma was collected and stored at −80°C. Insulin and C-peptide were measured by Rat/Mouse Insulin or C-peptide 2 ELISA Kit (cat. EZRMI-13K and EZRMCP2-21K, EMD Millipore, USA, resp.), according to the manufacturer's instructions. Insulin clearance was evaluated by C-peptide : insulin ratio, as previously described [16].

### 2.5. Islet Isolation and GSIS

Islets were isolated by collagenase digestion of the pancreas, as described by Boschero et al. 1995. For static incubations, groups of five islets were preincubated for 30 min at 37°C with 500 *μ*L of Krebs buffer (KBB) with the following composition: 115 mM NaCl, 5 mM KCl, 2.56 mM CaCl_2_, 1 mM MgCl_2_, 10 mM NaHCO_3_, 15 mM HEPES; supplemented with 5.6 mM glucose and 3 g bovine serum albumin (BSA) per liter; and equilibrated with a mixture of 95% O_2_-5% CO_2_ to provide pH 7.4. After, this medium was replaced with fresh buffer, and the islets were incubated for 1 h with 1 mL of KBB containing 5.6, 11.1, or 16.7 mM glucose. At the end of the incubation period, the supernatants were collected and maintained at −20°C. For islet insulin content, groups of five islets were collected, transferred to tubes containing 1 ml deionized water, and homogenized using a sonicator (Brinkmann Instruments, USA). The insulin was measured by RIA using human insulin radiolabelled with ^125^I as tracer, rat insulin as standard (Crystal Chem Inc., USA), and rat insulin antibody (donated by Dr. Leclerq-Meyer, Free University of Brussels, Belgium). The charcoal dextran method was used to separate free insulin from antibody-bound ^125^I insulin.

### 2.6. Western Blot

For Western blot analysis, liver samples from the mice were collected, snap-frozen in liquid nitrogen, and stored at −80°C, for subsequent protein extraction using a lysate buffer (10 mmol/L EDTA, 100 mmol/L Tris base, 100 mmol/L sodium pyrophosphate, 100 mmol/L sodium fluoride, 10 mmol/L sodium orthovanadate, 2 mmol/L phenylmethylsulphonyl fluoride, 1% Triton X-100, and 1 *μ*g/mL aprotinin). The Bradford method was performed to determine the protein concentration, using BSA as a standard. After, 50 *μ*g of the protein samples was homogenized with Laemmli buffer and boiled at 100°C during 5 min. These samples were resolved using 10% SDS-PAGE and electroblotted into nitrocellulose membranes. These membranes were blocked in 10 mmol/L Tris base, 150 mmol/L NaCl, and 0.25% (vol/vol) of Tween 20 (TBS buffer) containing 5% (wt/vol) BSA for 1 h at room temperature. Membranes were then incubated with primary antibodies (IDE, Abcam cat. ab32216; anti-GAPDH, Sigma cat. G9545) overnight at 4°C. The detection was performed by enhanced chemiluminescence (SuperSignal West Femto, Pierce Biotechnology Inc., Rockford, IL, USA) after incubation with horseradish peroxidase-conjugated secondary antibody. The bands were visualized using an Amersham Imager 600 (GE Healthcare Biosciences), and the intensities were quantified using ImageJ software (National Institutes of Health, Bethesda, MD, USA).

### 2.7. Statistical Analysis

The data are presented as the means ± SEM, and the differences were considered significant when *p* < 0.05. Comparisons were performed using a one-way ANOVA followed by Tukey's test. Tests were carried out using GraphPad Prism, version 5.0 for Windows (GraphPad Software Inc., San Diego, CA, USA). Sample size was determined taking into account the size effect. Bilateral statistic with a significance level of 5% and potency of 0.98 was used to rule out type II errors. Under these conditions, the recommended sample size required would be *n* = 5; however, we opted for a size of *n* = 6 as a safety measure.

## 3. Results

### 3.1. Diets and Vagal Denervation Characterization

First of all, we characterized the malnourished model, which showed reduced body weight and serum total proteins (Supplemental Figure 1 available online at https://doi.org/10.1155/2017/9652978). Then, we confirmed the efficiency of the high-fat diet used, since the mice fed on this diet became obese with augmented adiposity. We also confirmed that vagotomy reduced body weight and fat pads (Supplemental Figure 2) in addition to improved glucose tolerance and insulin sensitivity in obese mice induced only by high-fat diet (Supplemental Figure 3), a well-known effect of this surgery. Surprisingly, vagotomy did not alter the body weight and adiposity in the LP + HFvag mice (Table 1). The stomach weight was higher in the LP + HFvag compared with that in the LP + HF mice, confirming the efficiency of the vagotomy. The fasting glycemia and insulinemia were higher in the LP + HF compared with that in the LP mice. The fasting insulinemia, but not glycemia, was reduced in the LP + HFvag compared with that in the LP + HF mice (Table 1). However, we did not observe difference in the fed insulinemia comparing the LP + HF with the LP + HFvag group.

### 3.2. Vagotomy Improved Glucose Tolerance but Not Insulin Sensitivity

**Figure 1:**
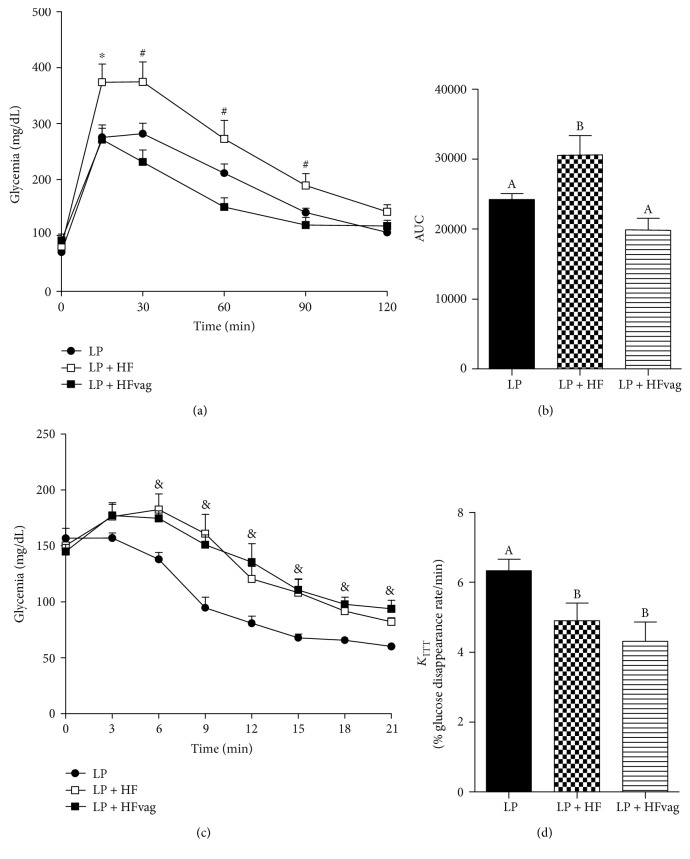
Vagotomy improved glucose tolerance but not insulin sensitivity. Blood glucose during the ipGTT (a) and its respective area under curve (AUC) (b). Blood glucose during ipITT (c) and glucose disappearance rate (*K*_ITT_) (d) in the LP, LP + HF, and LP + HFD mice. Data are means ± SEM from LP (*n* = 11–13); LP + HF (*n* = 8–10); LP + HFvag (*n* = 5-6). The symbols “∗” indicate significant differences between the LP and LP + HFvag compared to the LP + HF; “#,” between the LP + HF and LP + HFvag; and “&,” between the LP + HF and LP + HFvag compared to the LP. Different letters over the bars indicate significant differences. One-way ANOVA followed by Tukey's test. *P* < 0.05.

During ipGTT, the LP + HF mice had an increased glycemia (Figure 1(a)), indicating an impairment on glucose tolerance compared with the LP mice, as judged by the AUC (Figure 1(b)). Interestingly, vagotomy restored glucose tolerance in the LP + HFvag mice to the levels of those observed in the LP group, as observed in the AUC graph (Figure 1(b)). During ipITT (Figure 1(c)), the LP + HF mice displayed impairment on insulin sensitivity, compared with the LP group, as demonstrated by the *K*_ITT_ (Figure 1(d)). Although the vagotomy did not alter the insulin sensitivity, the LP + HFvag mice had an increased fed insulinemia (Table 1), which could explain the improved glucose tolerance in these mice.

### 3.3. Vagotomy Reduced GSIS in Isolated Pancreatic Islets

**Figure 2:**
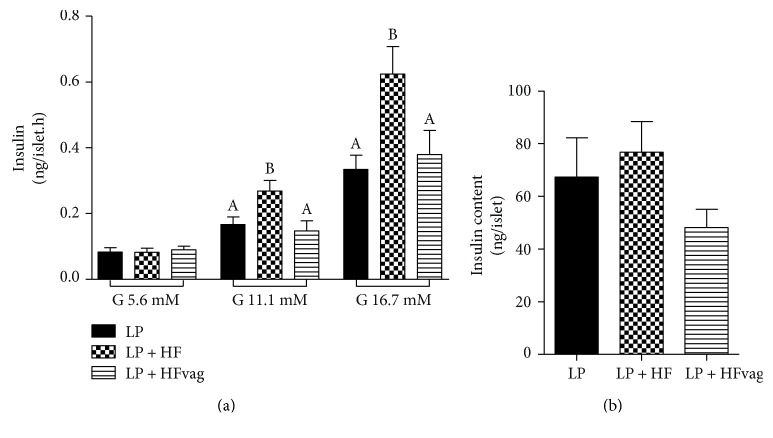
Vagotomy reduced GSIS in isolated pancreatic islets. Glucose-stimulated insulin secretion (a) and total insulin content (b) of islets from the LP, LP + HF, and LP + HFvag mice. Data are means ± SEM from the LP (*n* = 13); LP + HF (*n* = 8); and LP + HFvag (*n* = 8). Different letters over the bars indicate significant differences. One-way ANOVA followed by Tukey's test. *P* < 0.05.

To explain the higher insulinemia observed during ipGTT of the LP + HFvag mice, we accessed the GSIS in isolated pancreatic islets. At low glucose concentration (5.6 mM), insulin secretion of all groups was similar. However, at high glucose concentrations (11.1 and 16.7 mM), an increased insulin secretion in the islets from the LP + HF was observed, compared with the LP mice. The insulin secretion was lower in the islets from the LP + HFvag mice, reaching similar levels of those observed for the LP group (Figure 2(a)). The total insulin content of the islets in all groups was not significantly different (Figure 2(b)).

### 3.4. Vagotomy Reduced Insulin Clearance

**Figure 3:**
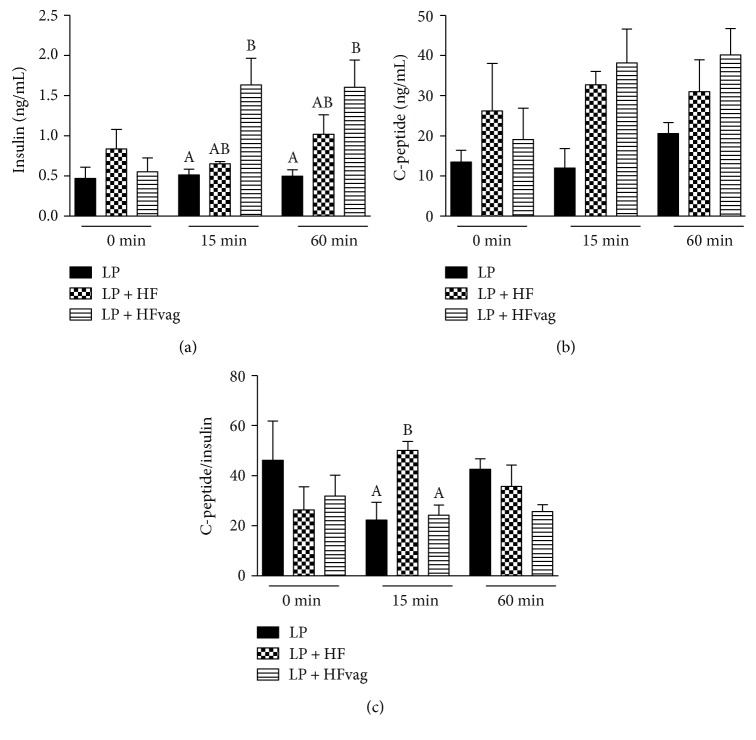
Vagotomy reduced insulin clearance. Plasma concentration of insulin (a), C-peptide (b), and C-peptide : insulin ratio (c) at 0, 15, and 60 min during ipGTT. Data are means ± SEM from the LP (*n* = 3–6); LP + HF (*n* = 3–5); and LP + HFvag (*n* = 4–6). Different letters over the bars indicate significant differences. One-way ANOVA followed by Tukey's test. *P* < 0.05.

The lower GSIS of the LP + HFvag mice did not justify the higher insulinemia found in these mice during the ipGTT (Figure 3(a)). Thus, we also evaluated the insulin clearance of these mice (measuring the C-peptide : insulin ratio). It is known that pancreatic *β* cells cosecrete insulin and C-peptide in a 1 : 1 ratio; however, the half-time of C-peptide is longer than that of insulin. Thus, an augmentation in the C-peptide : insulin ratio indicates an increased insulin clearance, as observed in the LP + HF, compared with the LP mice (Figure 3(c)). Interestingly, insulin clearance was reduced in the LP + HFvag mice, with a decreased C-peptide : insulin ratio, compared with the LP + HF group (Figure 3(c)), explaining the higher insulinemia of those mice during the ipGTT.

### 3.5. Vagotomy Reduced IDE Expression in the Liver of the LP Mice

**Figure 4:**
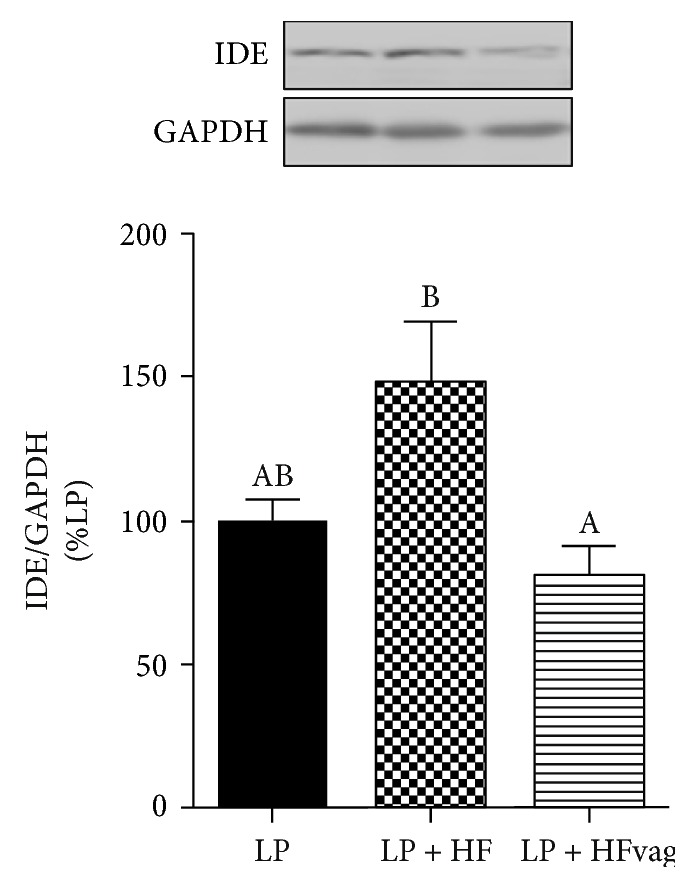
Vagotomy reduced IDE expression in the liver of the LP mice. IDE protein expression in the liver of the LP, LP + HF, and LP + HFvag mice. Data are means ± SEM from the LP (*n* = 6); LP + HF (*n* = 5); and LP + HFvag (*n* = 4). Different letters over the bars indicate significant differences. One-way ANOVA followed by Tukey's test. *P* < 0.05.

IDE is the most important protein involved in insulin clearance, a phenomenon that occurs mainly in the liver. Therefore, we evaluated IDE protein expression in the liver of mice. Corroborating the insulin clearance data, the LP + HF mice displayed higher IDE expression, compared with the LP group (Figure 4). The expression of this enzyme, in the liver of the LP + HFvag mice, was reduced, returning its values similar to those found in the LP mice (Figure 4).

## 4. Discussion

Previous studies have demonstrated that metabolic programmed mice, fed a low-protein diet during childhood followed by a control diet in adulthood, developed glucose intolerance, associated with augmented vagal activity [2]. Here, we performed vagotomy in mice kept on a low-protein followed by a high-fat diet to verify a possible role of the parasympathetic nervous system on their insulin-glucose homeostasis. We observed that vagotomy improved glucose tolerance of metabolic programmed mice by decreasing insulin clearance, which probably occurs through reduced expression of the liver IDE.

It is known that diet-induced obesity, in mice, also provokes glucose intolerance accompanied by increased insulin secretion, which compensates for the peripheral insulin resistance [17]. This phenomenon has also been detected in metabolic programmed mice fed on a low-protein diet in early life followed by regular diet [2] or high-fat diet [5, 18] during adulthood. Here, we confirmed these results with the LP + HF showing impaired insulin sensitivity and glucose tolerance, as well as an increased insulin secretion.

Increased vagal activity in obese and metabolic programmed mice has been associated with weight gain and higher insulin secretion, during the fed state [2, 19]. In addition, vagotomized obese rodents showed reduced body weight, due to decreased fat pads, associated with improved glucose tolerance, insulin sensitivity, and secretion. In fact, we confirmed that vagotomy was efficient to induce all these benefits, in the diet-induced obese mice (Figure S3). However, body weight and fat pads, as well as insulin action and secretion, were similar between the LP + HF and LP + HFvag mice. These results suggest that the improvement observed in glucose homeostasis, in the LP + HFvag mice, was independent of alteration in body composition. These findings confirmed that metabolic programming-induced obesity is more problematic than obesity per se, since the programmed mice did not display the well-known benefits from vagotomy, such as reducing in adiposity and body weight [13, 20–22]. The same scenario has been seen when metabolic programmed mice received taurine supplementation in an attempt to improve their glucose-insulin homeostasis [18].

Contrary to our findings, previous reports have demonstrated that vagotomy did not alter insulin clearance in lean pigs [23]. This suggests that vagotomy-induced reduction in insulin clearance is a phenomenon observed only in an obese mouse model, probably because they experienced malnutrition during early life. In fact, in nonprogramming obese rats, vagotomy also reduced insulin secretion but in contrast with our data, the insulinemia was reduced [24], which reinforces our idea that insulin clearance reduction induced by vagotomy may be dependent on metabolic programming. Although not evaluated, it seems that mice fed on a low-protein diet early in life followed by a regular diet in adulthood also develop decreased insulin clearance. This assumption is based on the observation that they display augmented plasma insulin concentration without an increase in glucose-stimulated insulin secretion [2]. However, the mechanism by which vagotomy reduced insulin clearance and liver IDE expression remains unclear.

Although reduced insulin clearance and liver IDE expression has been associated with insulin resistance and the development of glucose intolerance [25, 26], in early life, IDE KO mice showed higher plasma insulin concentration and improved glucose tolerance. However, maintaining high plasma insulin concentrations for an extended period led to insulin resistance and glucose intolerance in these mice at 6 months old [27]. This phenomenon seems to be caused by a negative feedback of the insulin pathway, orchestrated by the overstimulation of the proximal insulin cascade. Thus, the improvement of glucose tolerance, observed in the LP + HFvag mice, could be a transient effect, and these mice are susceptible to develop glucose intolerance and consequently T2D.

In conclusion, subdiaphragmatic vagotomy improves glucose tolerance in metabolic programmed mice fed on a low-protein diet early in life followed by exposure to a high-fat diet in adulthood. Vagotomy increases their plasma insulin concentration by reducing insulin clearance, a phenomenon probably due to diminished expression of liver IDE. However, it is necessary to keep in mind that strategies toward vagal activity inhibition, for the control of metabolic diseases, may jeopardize glucose tolerance over time.

## Supplementary Material

Supplemental Figure 1: Body weight (A) and serum total protein (B), of NP an LP groups. Data are means ± SEM from n = 6-8. The symbol ∗ indicate significant differences (P < 0.05). Student's t-test. Supplemental Figure 2: Body weight (A), perigonadal (B) and retroperitoneal fat pad (C), of NP, NP+HF and NP+HFvag groups. Data are means ± SEM from n = 6-8. Different letters over the bars indicate significant differences (P < 0.05). ANOVA one-way followed by Tukey's test. Supplemental Figure 3: Glucose tolerance and insulin sensitivity evaluated by ipGTT (A), AUC of ipGTT (B), ipiTT (C), KITT (D) in NP, NP+HF and NP+HFvag mice. Data are means ± SEM from n = 6-15. Different letters over the numbers indicate significant differences (P < 0.05). ANOVA one-way followed by Tukey's test.

## Figures and Tables

**Table 1:** Body, fat pads, and stomach weight, followed by blood glucose and plasma insulin concentration in the LP, LP + HF, and LP + HFvag mice.

	LP	LP + HF	LP + HFvag
Body weight (BW) (g)	23.6 ± 0.7^b^	27.6 ± 1.0^a^	27.4 ± 0.7^a^
Retroperitoneal fat pad (% BW)	0.6 ± 0.1^a^	1.2 ± 0.1^b^	0.9 ± 0.2^b^
Perigonadal fat pad (% BW)	1.5 ± 0.1^a^	2.3 ± 0.3^b^	1.9 ± 0.3^ab^
Stomach weight (% BW)	1.5 ± 0.2^a^	0.9 ± 0.1^a^	2.5 ± 0.4^b^
Serum total Protein (g/dL)	4.0 ± 0.7^b^	5.7 ± 0.3^a^	5.4 ± 0.3^ab^
Fasting glycemia (mg/dL)	55 ± 1^b^	70 ± 4^a^	67 ± 6^a^
Fasting insulinemia (ng/mL)	0.09 ± 0.01^a^	0.28 ± 0.06^b^	0.14 ± 0.07^a^
Fed insulinemia (ng/mL)	1.62 ± 0.13^a^	2.56 ± 0.26^b^	2.70 ± 0.24^b^

Data are means ± SEM from the LP (*n* = 6–13); LP + HF (*n* = 6–13); and LP + HFvag (*n* = 5–10). ^a,b^Significant differences (*P* < 0.05).
